# Real-time automatic detection of gynecological laparoscopic surgical instruments and exploration in surgical skills assessment application: a cross-sectional study

**DOI:** 10.1097/JS9.0000000000002699

**Published:** 2025-06-12

**Authors:** Huanyu Wei, Li Deng, Xueju Wu, Wenwei Tan, Yi Wu, Bin Yi, Yudi Li, Ruiwei Wang, Xiaolong Liang, Yin Chen, Hui Wang, Shuai Tang, Yanzhou Wang

**Affiliations:** a Department of Obstetrics and Gynecology, The First Affiliated Hospital of Army Medical University (Third Military Medical University), Chongqing, China; b Department of Obstetrics and Gynecology, No. 960 Hospital of the Joint Service Support Force of the Chinese People’s Liberation Army, Jinan, China; c Department of Digital Medicine, College of Biomedical Engineering and Medical Imaging, Army Medical University (Third Military Medical University), Chongqing, China; d Department of Anesthesiology, The First Affiliated Hospital of Army Medical University (Third Military Medical University), Chongqing, China; e Department of Obstetrics and Gynecology, The 958th Army Hospital of the Chinese People’s Liberation Army (958th Hospital), Chongqing, China; f Hunan Provincial Engineering Research Center for Gynecological Intelligent Diagnostic and Treatment Equipment Products, Hunan, China

**Keywords:** artificial intelligence surgery, cross-sectional study, deep learning, gynecological laparoscopic surgery, surgical instruments detection, surgical skill assessment

## Abstract

**Background:**

Automatic detection of surgical instruments is essential for artificial intelligence surgery. This study aimed to construct a large-scale dataset of gynecological laparoscopic surgical instruments based on real surgical scenarios, achieve high-precision real-time detection of surgical instruments, and explore their potential application in surgical skill evaluation.

**Materials and methods:**

This cross-sectional study collected 265 gynecological laparoscopic surgical videos from two medical centers for instrument detection. Videos were divided into training and testing sets in a 4:1 ratio, with 161 348 instrument instances extracted. The instruments were detected using Real-Time Models for Object Detection (RTMDet). The mean average precision, sensitivity, and F1 score served as evaluation metrics. External validation was conducted on an independent dataset from a third medical center. Additionally, we further compared the RTMDet with the state-of-the-art PP-YOLOE model on the same dataset. Furthermore, this study performed real-time tracking of instruments during the vaginal cuff suturing step of laparoscopic hysterectomy and compared the differences in kinematic data between proficient and non-proficient videos.

**Results:**

The mean average precision, sensitivity, and F1 score for nine types of surgical instruments were 91.75%, 94.29%, and 93.00%, respectively. External validation on the independent dataset demonstrated robust performance. In the comparison with PP-YOLOE, RTMDet demonstrated superior performance in all metrics. In the comparative analysis of kinematic data, the proficient group demonstrated significantly lesser path lengths and inter-quartile range, shorter moving times, and higher movement velocities for instruments used by both hands compared to the non-proficient group.

**Conclusions:**

This study established a large-scale, real scenario-based database of gynecological laparoscopic instruments. Using the RTMDet model, high-precision real-time detection and tracking of multiple instruments were achieved. Furthermore, this study identified several instrument kinematic metrics that can be used for surgical skill assessment, providing a reference for the objective quantification of the subjective Global Operative Assessment of Laparoscopic Skills.

## Introduction

Artificial intelligence (AI) has recently witnessed significant advancements, notably penetrating the field of medicine with remarkable achievements in imaging and pathological diagnosis^[1,2]^. However, its progress in the surgical field has been slower. Drawing parallels with autonomous driving technology, the ultimate goal for AI in surgery is fully automated surgical procedures^[3]^, wherein robotic systems can independently perform operations. However, this ambitious goal will need a step-by-step approach^[4]^.

In a 2022 white paper discussing the definitions of AI and autonomous actions in clinical surgery, six levels of advancement for automated surgical robots were defined: no autonomy, robot assistance, task autonomy, conditional autonomy, high autonomy, and full autonomy^[3]^. Currently, the evolution of surgical autonomy primarily resides at the level of robotic assistance^[5]^, where robots provide mechanical assistance with negligible autonomy. A key step toward greater autonomy is environmental perception^[6,7]^, including automated detection of surgical instruments and anatomical structures. Research into surgical tool detection could lay a solid foundation for recognizing more complex organs and tissues, thereby providing an ideal entry point for exploring the application of AI in surgery.

In the field of surgical instrument detection, computer vision-based methods have gained increasing attention due to their noninvasive nature and ability to achieve high recognition accuracy without instrument modification. The emergence of deep learning, along with representative models like Convolutional Neural Networks (CNNs), the YOLO series, and transformer-based architectures, has significantly advanced the accuracy of object recognition. Despite the advancements in computer vision-based methods, their practical application in surgical environments is still constrained by several critical limitations, particularly in dataset construction and algorithm design.

High-precision automated detection of surgical instruments primarily relies on high-quality data annotation and well-designed model architecture.^[8-10]^ Despite studies reporting satisfactory detection accuracy,^[11-13]^ several challenges remain in these two areas. First, regarding dataset construction, most studies depend on public datasets where images are composed of pre-selected images, often excluding blurred images, and typically sourced from a single medical institution. This can lead to overfitting, where a model performs exceptionally well on a specific dataset but poorly in real surgical environments^[14]^, limiting its practical application. Moreover, some instruments frequently used in gynecological laparoscopic surgery, such as myoma-grasping forceps, are not included in these public datasets, rendering them unusable for research in the automatic detection of gynecological laparoscopic instruments. Second, regarding algorithm design, detection accuracy and efficiency are the most critical indicators in its practical application. However, most current object detection algorithms fail to balance accuracy and efficiency, limiting their use in real surgical scenarios. The real-time multitask detection (RTMDet) model, proposed by Lyu *et al*. in 2022 and based on deep learning, has made significant progress in addressing this challenge^[15]^. However, its application has predominantly focused on the industrial field, with limited research in medical image detection.

Therefore, we aimed to retrospectively collect surgical video recordings and develop a dataset for gynecological laparoscopic instruments used in real-world scenarios, to automate the recognition, localization, and tracking of these instruments using a deep-learning model called RTMDet. Furthermore, we explored the potential of instrument detection to assess surgical skills, particularly during the critical phase of vaginal stump suturing in laparoscopic hysterectomy. This cross-sectional study has been reported in line with the Strengthening The Reporting Of Cohort Studies in Surgery (STROCSS) guidelines^[16]^.

## Material and methods

### Ethics statement

This study protocol was approved by the ethics committee of Army Medical University, Chongqing, China on April 28, 2024 (approval number: KY2024147), and was conducted in accordance with the principles of the Declaration of Helsinki. As this was a retrospective study, the requirement for informed consent was waived. We followed the STROBE reporting guidelines. The work was reported in line with the STROCSS criteria^[16]^.

**HIGHLIGHTS**

- A large-scale gynecological laparoscopic surgical instrument database was developed.
- High-precision real-time multiple instrument detection and tracking were achieved.
- Several instrument kinematic metrics were identified for surgical skill assessment, providing an objective quantification reference for the subjective Global Operative Assessment of Laparoscopic Skills criteria.

### Preparation of the surgical instrument dataset

This cross-sectional study included 265 surgical videos from the obstetrics and gynecology departments of The First Affiliated Hospital of Army Medical University and The 958th Army Hospital of the Chinese People’s Liberation Army. These videos were gathered from 1 March 2022 to 31 July 2022. The inclusion criterion was surgical videos of traditional laparoscopic procedures performed for any gynecological disease. The video resolutions were 1920 × 1080, 720 × 576, and 720 × 480 pixels (frame rate: 24–30 fps). Robotic surgery, as well as incomplete or improperly formatted video recordings, were excluded.

The videos were subsequently divided into training and testing datasets in a 4:1 ratio. Considering the high similarity between surgical instruments and background information in consecutive frames, we selectively extracted frames at 8-s intervals to avoid data redundancy and reduce ineffective training samples. Irrelevant scenes and frames lacking the target surgical instruments were removed manually.

**Figure 1.**
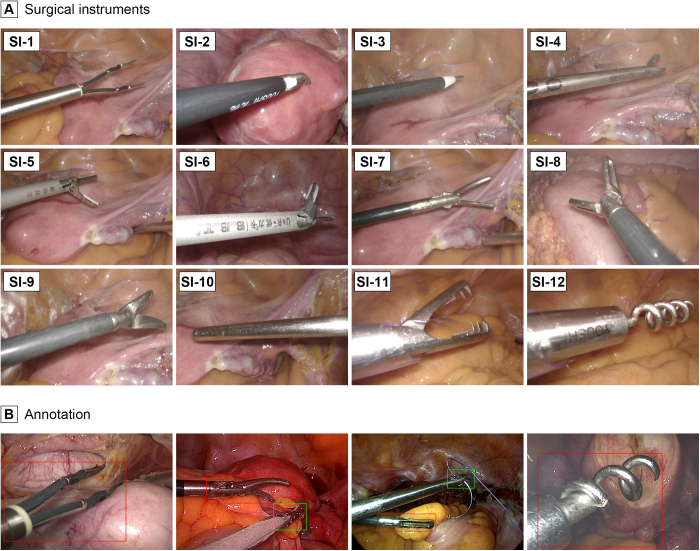
Illustrations and annotation methods of surgical instruments. Panel A shows the illustrations of 12 surgical instruments, with SI-5 and SI-6, as well as SI-7 and SI-8, consolidated into single categories for the study. Panel B demonstrates the manual annotation method for surgical instruments.

Twelve surgical instruments were incorporated (Fig. 1A), including two types of bipolar forceps (SI-1, 2), a monopolar hook electrode (SI-3), a needle holder (SI-4), two types of ultrasound knives (SI-5, 6), two types of grasping forceps (SI-7, 8), scissors (SI-9), suction irrigation tubes (SI-10), myoma-grasping forceps (SI-11), and a myoma drill (SI-12). Notably, to reduce model complexity and align with clinical requirements, the two types of ultrasound knives and grasping forceps were consolidated into singular categories based on their similar appearances and functionalities. Five computer researchers annotated the images with bounding boxes under the guidance of two gynecologists (Fig. 1B).

### Preparation of the external validation dataset

Most existing open-source public datasets mainly cover robotic surgical instruments or those for laparoscopic cholecystectomy, which differ significantly in shape from the instruments included in this study. Therefore, we collected videos of gynecological laparoscopic surgeries from No. 960 Hospital of the Joint Service Support Force of the Chinese People’s Liberation Army for external validation. The data processing methods were consistent with those used in the preparation of the surgical instrument dataset. During the manual annotation phase, all surgical instruments in the external validation set were labeled by a single gynecologist.

### Detection of surgical instruments

**Figure 2.**
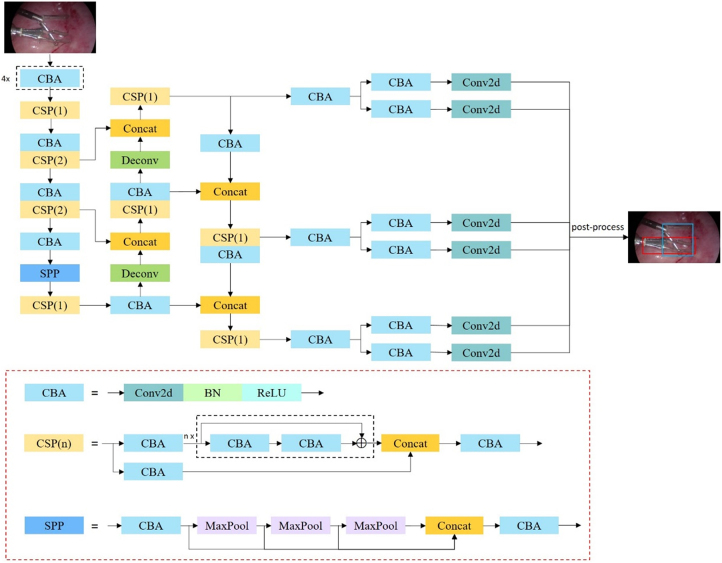
Diagram of the network structure. Conv2d, Convolution Layer; BN, Batch Normalization; SiLU, Sigmoid Linear Unit; MaxPool, Max Pooling; CBA, Convolutional Block Attention; ConCat, Concat Layer; CSP, Cross Stage Partial; SPP, Spatial Pyramid Pooling; ⨁, Element-wise Sum; Deconv, Deconvolution.

A deep learning-based object detection algorithm was used to detect the surgical instruments. The model adopted was developed based on RTMDet by incorporating specific modifications into the original framework. The primary modification involved pruning optimization^[17]^, which was dynamically adjusted during the experimental process to ensure the model met the frame rate requirements for practical applications. The constructed network comprises three major components: a modified backbone network based on CSPNeXt^[18]^, a feature-augmentation module using PANet^[19]^, and a detection head (Fig. 2). The backbone network primarily generates downsampled feature maps with factors of 8, 16, and 32. The feature-augmentation module enhances these images by integrating feature maps from various hierarchical levels, and the detection head interprets these maps to generate predictive outcomes, including confidence scores, classification labels, and coordinates of the detected instruments. Predictions by the model were considered valid only if the generated confidence scores exceeded a threshold of 0.2. After detection, results were refined through post-processing to produce the final bounding boxes. Specifically, we applied non-maximum suppression (NMS) with a threshold of 0.3 to filter overlapping bounding boxes and retain the most accurate predictions. Model pre-training was accomplished using the Microsoft Common Objects in Context (MS COCO) dataset^[20]^.

For data management, the dataset comprised images at three different resolutions. However, only the images resized to 960 × 544 pixels were used in the inference stage. This resolution was finalized by downsampling or resizing the original input, followed by strategic zero padding along the inferior and right margins. Before network submission, each RGB image was individually standardized. Specifically, for each pixel in the three channels (red, green, and blue), the mean values (123.675 for red, 116.28 for green, and 103.53 for blue) were subtracted, and then the results were divided by variance (58.395 for red, 57.12 for green, and 57.375 for blue). Data augmentation was implemented as an online augmentation scheme and applied to each input batch. To ensure the reproducibility and reference value of the test results, data augmentation was not performed on the test set. The computational framework for training employed eight NVIDIA Tesla V100 GPUs with a batch size of 256, using the AdamW optimizer with an initial learning rate of 0.004. A detailed comparison of the hyperparameters used in this study with the original RTMDet implementation is provided in Supplemental Digital Content, Table 1, available at: http://links.lww.com/JS9/E332. Specific details of the data augmentation methodologies are provided in Supplemental Digital Content, Table 2, available at: http://links.lww.com/JS9/E333. In total, 300 epochs were conducted to derive the final model, which was subsequently implemented in an NVIDIA Tesla V100 within the inference environment. Additionally, we further compared the RTMDet with the state-of-the-art PP-YOLOE model on the same surgical instrument dataset.

### Preparation of the vaginal stump suturing dataset

Eleven surgical video recordings of laparoscopic hysterectomies distinct from the surgical instrument dataset were included in the suturing dataset. Segments of the vaginal stump sutures were extracted manually. Subsequently, three clinical experts independently assessed the videos based on the Global Operative Assessment of Laparoscopic Skills (GOALS)^[21]^, which evaluates laparoscopic surgery performance according to the following five fundamental facets: depth perception, bimanual dexterity, efficiency, tissue handling, and autonomy. Each aspect was scored on a rating of 1–5. Given the impracticality of the experts in ascertaining the absence or presence of auxiliary guidance from another physician, the autonomy criterion was omitted. Consequently, the possible score for each video ranged from 4 to 20. The average scores were calculated, and the videos were classified as proficient or non-proficient using a cutoff score of 12. This assessment was performed in a single-blind manner to avoid bias related to the surgeon’s identity.

### Tracking of surgical instruments

Employing the object detection algorithm, the categories and bounding boxes of the surgical instruments present in the videos of vaginal stump suturing were predicted frame-by-frame. It is important to note that vaginal stump suturing is primarily performed using grasping forceps and needle holders. In special cases, such as when a suction irrigation tube is needed to clear a blood-contaminated surgical field, other instruments may appear. However, in the analyzed surgical videos, only these two instruments were present. During prediction, they might be incorrectly classified as other instruments. To avoid trajectory interruptions from such mispredictions, we retained only the detection boxes corresponding to these two types of surgical instruments. Subsequently, the centroid coordinates of the predicted bounding boxes were computed and recorded. If an instrument was detected in successive frames, a line was drawn linking the pertinent points, thereby generating a trajectory that delineates the instrument’s motion. The length of these trajectories embodies the path length traversed by the instruments, quantified in pixels, whereas the comprehensive tally of points disseminated along the trajectory signifies the moving time of the instruments, quantified in frames. Consequently, the quotient derived by dividing the instrument’s path length by its moving time was defined as the movement velocity. Given the complexity of motion trajectories during the entire vaginal stump suturing phase, the process was methodologically decomposed based on individual stitches. The start point of a single stitch was defined as when the needle initially contacted the vaginal stump tissue, and the endpoint was defined as when the needle contacted the tissue again for the next stitch.

### Evaluation metrics

Precision, sensitivity (recall), and F1 score served as metrics to evaluate the predictive capabilities of the models, which are characterized by the quantities of true positives (TP), false positives (FP), and false negatives (FN). High precision indicates a lower risk of mistakenly identifying instruments. High sensitivity signifies a reduced risk of missed identifications. The F1 score, which is the harmonic mean of precision and sensitivity, effectively balances the performance of both measures. The calculation formulas are as follows:

$$Precision=\frac{TP}{TP+FP}$$

$$Sensitivity=\frac{TP}{TP+FN}$$

$$F1=2\cdot \frac{Precision\;.\;Sensitivity}{Precision+Sensitivity}$$

Prediction was defined as a TP when the intersection over union (IoU) is >0.3; otherwise, it was defined as an FN. An FP was defined as a predicted bounding box without a corresponding ground-truth bounding box. IoU was utilized to assess the accuracy of the instrument localization by calculating the ratio of the intersection to the union of the predicted and actual localization boxes. The calculation formula is as follows:

$$IoU=\frac{area\left(P\right)\cap \;area(GT\;)}{area\left(P\right)\cup \;area(GT\;)}$$

where *P* indicates the predicted bounding boxes, and *GT* indicates the manually annotated ground-truth bounding boxes.

### Statistical analyses

Statistical analyses were performed using the Statistical Package for the SPSS (version 26.0). Continuous variables are described as median with interquartile range and were subjected to comparative analysis using the Mann–Whitney *U* test. Statistical significance was set at *P* < 0.05.

## Results

### Detection of surgical instruments

**Figure 3.**
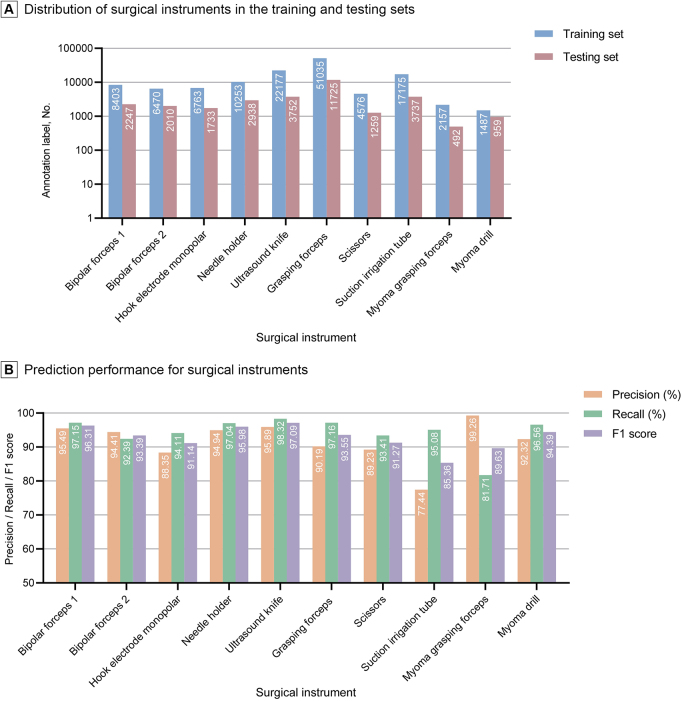
Quantities and prediction performance of surgical instruments. Panel A depicts the distribution of surgical instruments in the training and testing sets. Panel B shows the prediction performance for each surgical instrument.

**Figure 4.**
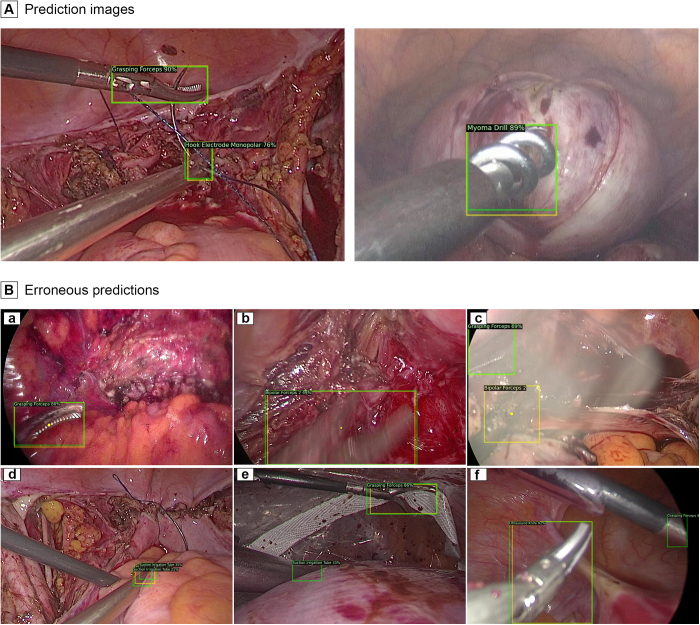
Prediction images and erroneous predictions of the model. Yellow box indicates the ground-truth box, while green indicates the predicted one. Panel A shows the prediction images. Panel B shows the instances of erroneous predictions. Yellow dots indicate instances of missed detection of instruments. Incorrect category prediction at correct location is classified as a missed detection.

A total of 265 surgical videos from the two medical centers were included. The extraction process resulted in 95 088 images capturing 161 348 instances of 12 surgical instruments, including 130 496 and 30 852 instances in the training and testing sets, respectively (Fig. 3A). The performance of each surgical instrument in the testing set is shown in Figure 3B. The ultrasonic knife achieved the highest F1 score of 97.09%, whereas the F1 score of the suction irrigation tube dropped notably to 85.36%. The model generally achieved a mean average precision (mAP) of 91.75%, a sensitivity of 94.29%, and an F1 score of 93.00% in the testing set. Instances displaying the automatic detection of surgical instruments are depicted in Figure 4A, demonstrating their capability to identify and locate multiple surgical instruments within an image. The adopted model achieved real-time detection of surgical instruments at 30 fps, as illustrated in Supplemental Digital Content, Video 1, available at: http://links.lww.com/JS9/E337.

During the construction of the external validation dataset, the data were sourced from a single medical center, resulting in limited coverage of surgical instrument types. Specifically, data for bipolar forceps 2 and myoma drill were not collected. In the external validation set, a total of 1401 images were included, capturing 2564 targets. On the external validation dataset, the RTMDet achieved an mAP of 87.57%, a sensitivity of 93.37%, and an F1 score of 90.37%. The detailed predictive performance for each surgical instrument is provided in Supplemental Digital Content, Table 3, available at: http://links.lww.com/JS9/E334.

Specific prediction errors manifested under the following circumstances: (1) the model missed the detection of bipolar forceps 2 and incorrectly predicted it as grasping forceps due to the similarity in their external shapes (Fig. 4Ba); (2) suboptimal image quality leading to missed detection and prediction errors, including conditions such as fog, motion blurring, and excessively dark scenes (Fig. 4Bb, c); (3) the emergence of numerous identical predictions for a single instrument is a phenomenon particularly prevalent in suction irrigation tubes (Fig. 4Bd); (4) erroneous predictions of suction irrigation tubes under conditions in which the tips of the surgical instruments are obscured, rendering only the shaft visible (Fig. 4Be); (5) if the tip of an instrument was severely occluded, it was customarily omitted from the annotations. Nonetheless, the use of random cropping during the training process may induce the model to generate prediction boxes for instruments that are only partially discernible (Fig. 4Bf).

We have further compared the RTMDet model with the state-of-the-art PP-YOLOE model on the same surgical instrument dataset. The PP-YOLOE achieved a mAP of 90.66%, a sensitivity of 93.05%, and an F1 score of 91.78% in the testing set. The experimental results showed that RTMDet outperformed PP-YOLOE in all three metrics, fully validating the superiority of RTMDet in the task of automatic surgical instrument detection. The specific comparison data are shown in Table 1.

**Table 1:** Comparison of RTMDet with PP-YOLOE

Instrument	RTMDet			PP-YOLOE		
	Precision (%)	Sensitivity (%)	F1 score (%)	Precision (%)	Sensitivity (%)	F1 score (%)
Bipolar forceps 1	95.49	97.15	96.31	93.21	96.33	94.74
Bipolar forceps 2	94.41	92.39	93.39	92.55	93.12	92.83
Hook electrode monopolar	88.35	94.11	91.14	89.11	90.35	89.73
Needle holder	94.94	97.04	95.98	92.77	95.13	93.94
Ultrasound knife	95.89	98.32	97.09	96.32	97.55	96.93
Grasping forceps	90.19	97.16	93.55	89.03	95.49	92.15
Scissors	89.23	93.41	91.27	89.91	94.15	91.98
Suction irrigation tube	77.44	95.08	85.36	79.01	90.76	84.48
Myoma grasping forceps	99.26	81.71	89.63	91.11	83.88	87.35
Myoma drill	92.32	96.56	94.39	93.55	93.74	93.64
AVG	91.75	94.29	92.81	90.66	93.05	91.78

### Tracking of surgical instruments

**Figure 5.**
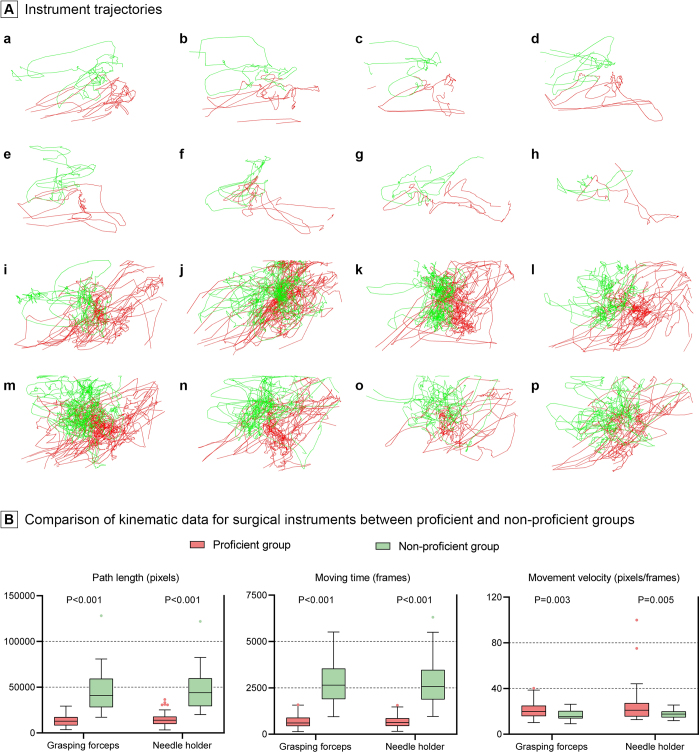
Kinematic data of surgical instruments. Panel A shows the instrument trajectories. (a–h) Motion trajectories of each stitch in the proficient group. (i–p) Motion trajectories in the non-proficient group. Within these images, green lines indicate the trajectories of the grasping forceps, while red lines indicate the trajectories of the needle holder. Panel B shows the comparison of the kinematic data of each surgical instrument per stitch between the proficient and non-proficient groups.

Eleven vaginal stump suturing videos were included for surgical skill analysis. Three clinical experts evaluated the surgical videos according to the GOALS criteria. Finally, seven videos were deemed proficient, and the remainder were labeled non-proficient. Detailed scoring results are shown in Supplemental Digital Content, Table 4, available at: http://links.lww.com/JS9/E335. The stitch counts in the proficient and non-proficient groups were 62 and 28 stitches, respectively. Subsequently, the motion trajectories of the instruments were generated. Figure 5Aa–h indicates the motion trajectories of each stitch in the proficient group, whereas i–p indicates those in the non-proficient group. The proficient group exhibited more streamlined motion trajectories and a marked similarity in the trajectories of each stitch compared with the non-proficient group. Furthermore, a detailed computation was conducted to determine the variances in the kinematic data. The proficient group demonstrated significantly lesser path lengths and inter-quartile range, shorter moving times, and higher movement velocities for grasping forceps and needle holders than the non-proficient group (Fig. 5B). The detailed kinematic data are presented in Supplemental Digital Content, Table 5, available at: http://links.lww.com/JS9/E336.

## Discussion

To the best of our knowledge, this is the largest-scale study on the automatic detection of laparoscopic surgical instruments and is the first application of RTMDet in the medical field. We developed a large-scale database of gynecological laparoscopic instruments and achieved high-precision real-time detection of multiple surgical instruments. Furthermore, we analyzed the differences in kinematic characteristics between videos labeled as proficient or non-proficient. The initial exploration affirmed the potential value of instrument detection for assessing surgical skills.

In object detection tasks, there is often a delicate balance to be maintained between accuracy and efficiency, which can be described as enhancing the precision often comes at the cost of reduced inference speed or increased consumption of computational resources. However, RTMDet achieves a remarkable balance between high accuracy and low latency. This is accomplished by improving efficiency and precision using large-kernel depth-wise convolution and applying soft labels in dynamic label assignments. Additionally, the integration of advanced training techniques further amplifies its performance. These improvements make RTMDet particularly suitable for medical applications that require real-time analysis and decision-making. In this study, by employing the RTMDet algorithm, all three metrics (precision, sensitivity, and F1 score) surpassed the 90% threshold. Xu *et al*. compared PP-YOLOE with several mainstream object detection models, such as YOLO series and EfficientDet, and demonstrated that PP-YOLOE achieved the best detection performance on the COCO dataset^[22]^. Based on this finding, we further compared the RTMDet model with the state-of-the-art PP-YOLOE model on the same surgical instrument dataset. The experimental results showed that RTMDet outperformed PP-YOLOE in accuracy, recall, and F1 score, fully validating the superiority of RTMDet in the task of automatic surgical instrument detection. For comparative analysis, we conducted a review of similar studies. Yamazaki *et al*. utilized YOLOv3 to detect 14 surgical instruments, achieving an average precision of 87% and sensitivity of 83%^[13]^. Vardazaryan *et al*. evaluated several architectures and achieved an mAP of 87.4% and a sensitivity of 88.8%^[23]^. Although different datasets were used, and direct comparison might have inherent limitations, surpassing 90% in accuracy, sensitivity, and F1 score sufficiently demonstrated the unique advantages of RTMDet in surgical instrument detection tasks.

While the RTMDet demonstrated exceptional performance across most surgical instruments, the suction irrigation tube exhibited a relatively lower F1 score of 85.36%, primarily attributed to a lower precision rate of 77.44%. This indicates that 22.56% of the predicted samples were FP, suggesting challenges in distinguishing the suction irrigation tube from other instruments. Specifically, the model exhibited a tendency to misclassify the shafts of other instruments as the suction irrigation tube, a phenomenon likely attributable to visual similarities in the shaft portion of these instruments. This issue was further compounded by the complex visual environment inherent to surgical settings, where instruments often overlap or were partially occluded, thereby increasing the likelihood of misclassification. Future work needs to explore strategies to address these challenges, including targeted data augmentation and the adoption of lightweight architectures, with the aim of enhancing the model’s ability to accurately distinguish the suction irrigation tube from other surgical instruments.

Some drawbacks of mainstream public datasets include single-source data and limited coverage of surgical scenarios. To enhance the model’s generalization and predictive performance, we adopted several critical measures during dataset construction. First, surgical videos from two medical centers were included in the dataset, covering almost all types of gynecologic laparoscopic instruments, which ensured the broad applicability of the model. Second, we established a large-scale dataset by collecting numerous surgical videos. The increase in data volume allowed the model to acquire a broader range of surgical scenarios and instrument characteristics during training, thereby improving its performance across varied surgical environments. Finally, during manual annotation, we provided annotations even when the images were blurred or fogged, provided that the instrument type could be identified by the annotators. The meticulous annotation strategy effectively enhanced the model’s robustness, enabling it to perform excellently in complex surgical scenarios.

In this study, we achieved high-precision real-time detection of surgical instruments and conducted corresponding application tests, further demonstrating its value in surgical skill assessment. Previous studies have demonstrated that surgical skills profoundly influence patient outcomes, particularly in complicated surgical procedures.^[24-26]^ Video-based assessment can provide surgeons with comprehensive feedback, improving their surgical capabilities^[27]^. Traditional video-based assessments typically rely on retrospective review of surgical videos by experts, which is time-consuming and may suffer from unavoidable biases^[28]^. Consequently, there is a pressing need for an objective and automated assessment tool to improve the current situation. For example, Jin *et al*. analyzed timelines, heat maps, and trajectories of surgical instruments during laparoscopic cholecystectomy, reporting significant differences in tool utilization, operational focus areas, and movement patterns among surgeons with different competencies^[29]^. However, their findings lacked quantification and thus failed to standardize surgical skill evaluation metrics. As is well-known, suturing is a fundamental skill for surgeons. Specifically, the procedural complexity of vaginal cuff suturing during laparoscopic hysterectomy exhibits minimal variation among patients. Consequently, the current study utilized vaginal cuff suturing as a focal point, generated trajectories of surgical instruments, and explored the differences in surgical videos labeled as proficient or non-proficient. Through a comparative analysis of videos of vaginal stump suturing performed by surgeons with different skill levels, we discovered that the proficient group exhibited reduced path length and moving time, increased kinematic velocity, and reduced interquartile range for each suture, which correspond to bimanual dexterity, efficiency, tissue handling, and depth perception outlined in the GOALS. Therefore, these subjective indicators can be described using quantifiable metrics. This imparts greater significance to our research and offers the potential to provide valuable feedback to surgical teams in the future, thereby playing a crucial role in enhancing surgical education.

Beyond its role in surgical skill assessment, the analysis of surgical instrument motion trajectories holds broader implications for optimizing surgical workflows and enhancing training methodologies. By analyzing these motion trajectories, it is possible to optimize surgical workflow design and provide data support for standardizing surgical techniques. Furthermore, real-time alerts can be triggered when surgical instruments significantly deviate from the optimal trajectories, enabling young surgeons to adjust their operations promptly and reduce surgical risks. Additionally, post-operative analysis of instrument motion trajectories can identify patterns of efficiency and inefficiency in surgical operations. This helps young surgeons pinpoint areas for improvement and achieve targeted skill enhancements. These technological advancements can transform surgical training from traditional experiential teaching to an intelligent and standardized approach, ultimately leading to significant improvements in surgical training efficiency.

Our study has some limitations. First, although the model proposed has been validated using an external dataset from another medical center, it is still in the proof-of-concept stage. Ideally, validation should be performed on a publicly available dataset to ensure broader generalizability. However, existing open-source datasets primarily focus on surgical instruments used in robotic surgeries or laparoscopic cholecystectomies, which differ significantly in shape from the instruments included in our study. Therefore, we had to rely on our own constructed external validation set. Second, all the surgical videos in this study were in a two-dimensional format. Consequently, the Z-axis was not included in the trajectory, which may have led to systematic errors. Disparate distances between the camera and the vaginal stump across videos can lead to systematic errors as well. Third, the detection performance of the suction irrigation tube was less satisfactory than that of other instruments. Future studies could address this issue by introducing lightweight architectures to improve the model’s ability to distinguish between instruments. Furthermore, although this study identified several potential indicators for assessing surgical skills, we believe that there are still numerous undiscovered differential indicators within kinematic metrics. Finally, the current study’s grouping design may overlook intermediate skill-level surgical cases, leading to extreme differences in results that may not fully reflect the continuity and diversity of skill levels in actual clinical practice. In future studies, it is necessary to further refine the grouping into expert, intermediate, and novice categories. This will help validate the applicability and sensitivity of these kinematic metrics across different skill levels, thereby providing a more comprehensive understanding of the complexity of surgical skill assessment and developing more universal evaluation methods.

## Conclusion

We developed a large-scale gynecological laparoscopic surgical instrument database based on real surgical scenarios, with detailed annotations of the positions and types of surgical instruments. For the first time, we applied the deep learning-based RTMDet to achieve high-precision real-time detection and tracking of multiple instruments within this database. In comparison with the state-of-the-art PP-YOLOE, RTMDet has shown superior performance, further confirming its effectiveness in the task of automatic surgical instrument detection. Furthermore, we identified key differences in the kinematic data of instruments between surgical videos of varying proficiency levels. These differences will aid in evaluating surgical skills and providing timely feedback to surgical teams, which may play a significant role in surgical education.

## Data Availability

For the identification of surgical instruments, we have invested significant effort in the manual annotation of surgical instruments. Considering the potential for further research using this dataset, we are currently not sharing the original data and source code. However, we are open to collaboration and can discuss data sharing on a case-by-case basis. Please contact the corresponding author for more information.

## Research registration unique identifying number (UIN)

Chictr.org Identifier: ChiCTR2400085622.

## Data access, responsibility, and analysis

Dr. Yanzhou Wang and Dr. Shuai Tang had full access to all the data in the study and take full responsibility for the integrity of the data and the accuracy of the data analysis.

## References

[1] Chamberlin J Kocher MR Waltz J. Automated detection of lung nodules and coronary artery calcium using artificial intelligence on low-dose CT scans for lung cancer screening: accuracy and prognostic value. BMC Med 2021;19:55. 33658025 10.1186/s12916-021-01928-3 PMC7931546

[2] Bera K Schalper KA Rimm DL Velcheti V Madabhushi A. Artificial intelligence in digital pathology – new tools for diagnosis and precision oncology. Nat Rev Clin Oncol 2019;16:703–15. 31399699 10.1038/s41571-019-0252-y PMC6880861

[3] Andrew AG Frank A Konrad K. White paper: definitions of artificial intelligence and autonomous actions in clinical surgery. Art Intel Surg 2022;2:93–100.

[4] Yang GZ Cambias J Cleary K. Medical robotics – regulatory, ethical, and legal considerations for increasing levels of autonomy. Sci Rob 2017;2:eaam8638. 10.1126/scirobotics.aam8638 33157870

[5] Vasey B Lippert KAN Khan DZ. Intraoperative applications of artificial intelligence in robotic surgery: a scoping review of current development stages and levels of autonomy. Ann Surg 2023;278:896–903. 36177855 10.1097/SLA.0000000000005700 PMC10631501

[6] Li C Zhang G Zhao B. Advances of surgical robotics: image-guided classification and application. Natl Sci Rev 2024;11:nwae186. 39144738 10.1093/nsr/nwae186 PMC11321255

[7] Fiorini P Goldberg KY Liu Y Taylor RH. Concepts and trends in autonomy for robot-assisted surgery. Proc IEEE Inst Electr Electron Eng 2022;110:993–1011. 35911127 10.1109/JPROC.2022.3176828 PMC7613181

[8] Wang Y Sun Q Liu Z Gu L. Visual detection and tracking algorithms for minimally invasive surgical instruments: a comprehensive review of the state-of-the-art. Rob Autom Syst 2022;149:103945.

[9] Rueckert T Rueckert D Palm C. Methods and datasets for segmentation of minimally invasive surgical instruments in endoscopic images and videos: a review of the state of the art. Comput Biol Med 2024;169:107929. 38184862 10.1016/j.compbiomed.2024.107929

[10] Rodrigues M Mayo M Patros P. Surgical tool datasets for machine learning research: a survey. Int J Comput Vis 2022;130:2222–48.

[11] Alshirbaji TA Jalal NA Mller K. Surgical tool classification in laparoscopic videos using convolutional neural network. Curr Dir Biomed Eng 2018;4:407–10.

[12] Twinanda AP Shehata S Mutter D Marescaux J de Mathelin M Padoy N. EndoNet: a deep architecture for recognition tasks on laparoscopic videos. IEEE Trans Med Imag 2017;36:86–97. 10.1109/TMI.2016.2593957 27455522

[13] Yamazaki Y Kanaji S Matsuda T. Automated surgical instrument detection from laparoscopic gastrectomy video images using an open source convolutional neural network platform. J Am Coll Surg 2020;230:725–732.e1. 32156655 10.1016/j.jamcollsurg.2020.01.037

[14] Kitaguchi D Takeshita N Hasegawa H Ito M. Artificial intelligence-based computer vision in surgery: recent advances and future perspectives. Ann Gastroenterol Surg 2022;6:29–36. 35106412 10.1002/ags3.12513 PMC8786689

[15] Lyu C Zhang W Huang H. RTMDet: an empirical study of designing real-time object detectors. arXiv 2022:2212.07784.

[16] Agha RA Mathew G Rashid R. Revised strengthening the reporting of cohort, cross-sectional and case-control studies in surgery (STROCSS) guideline: an update for the age of artificial intelligence, Premier J Sci 2025:100081.

[17] Sunil V Salem A. Methods for pruning deep neural networks. arXiv. 2020:2011.00241.

[18] Zhang J Zhang J Zhou K Zhang Y Chen H Yan X. An improved YOLOv5-based underwater object-detection framework. Sensors (Basel) 2023;23. 10.3390/s23073693 PMC10099368 37050753

[19] Liu S Qi L Qin H Shi J Jia J. Path aggregation network for instance segmentation. arXiv 2018:1803.01534.

[20] Lin T-Y Maire M Belongie S. Microsoft COCO: common objects in context. arXiv 2015:1405.0312.

[21] Vassiliou MC Feldman LS Andrew CG. A global assessment tool for evaluation of intraoperative laparoscopic skills. Am J Surg 2005;190:107–13. 15972181 10.1016/j.amjsurg.2005.04.004

[22] Xu S Wang X Lv W. PP-YOLOE: an evolved version of YOLO. arXiv. 2022:2203.16250.

[23] Vardazaryan A Mutter D Marescaux J Padoy N. Weakly-supervised learning for tool localization in laparoscopic videos. arXiv 2018:1806.05573.

[24] Birkmeyer JD Finks JF O’Reilly A. Surgical skill and complication rates after bariatric surgery. N Engl J Med 2013;369:1434–42. 24106936 10.1056/NEJMsa1300625

[25] Curtis NJ Foster JD Miskovic D. Association of surgical skill assessment with clinical outcomes in cancer surgery. JAMA Surg 2020;155:590–98. 32374371 10.1001/jamasurg.2020.1004 PMC7203671

[26] Ichikawa N Homma S Funakoshi T. Impact of technically qualified surgeons on laparoscopic colorectal resection outcomes: results of a propensity score-matching analysis. BJS Open 2020;4: 486–98. 32207580 10.1002/bjs5.50263 PMC7260420

[27] Yanik E Schwaitzberg S De S. Deep learning for video-based assessment in surgery. JAMA Surg 2024;159:957–58. 38837128 10.1001/jamasurg.2024.1510

[28] Hung AJ Liu Y Anandkumar A. Deep learning to automate technical skills assessment in robotic surgery. JAMA Surg 2021;156: 1059–60. 34524401 10.1001/jamasurg.2021.3651

[29] Jin A Yeung S Jopling J. Tool detection and operative skill assessment in surgical videos using region-based convolutional neural networks. arXiv 2018:1802.08774.

